# Divergence Between Informant and Self-Ratings of Activities of Daily Living Impairments in Parkinson’s Disease

**DOI:** 10.3389/fnagi.2022.838674

**Published:** 2022-02-11

**Authors:** Sara Becker, Susanne Solbrig, Katja Michaelis, Bettina Faust, Kathrin Brockmann, Inga Liepelt-Scarfone

**Affiliations:** ^1^Department of Neurodegenerative Diseases, Hertie Institute for Clinical Brain Research, University of Tübingen, Tübingen, Germany; ^2^Department of Psychology, Faculty of Arts, University of Calgary, Calgary, AB, Canada; ^3^German Center for Neurodegenerative Diseases (DZNE), University of Tübingen, Tübingen, Germany; ^4^Studienzentrum Stuttgart, IB Hochschule für Gesundheit und Soziales, Stuttgart, Germany

**Keywords:** activities of daily living, caregiver, cognition, Functional Activities Questionnaire, Parkinson’s Disease, self-ratings, informant-ratings

## Abstract

**Objective:**

To examine the agreement between self- and informant-reported activities of daily living (ADL) deficits in Parkinson’s Disease (PD) patients, and to examine factors influencing ADL ratings.

**Background:**

In PD, the loss of functional independence is an important outcome of disease progression. The valid assessment of ADL function in PD is essential, but it is unclear to what extent informants’ and patients’ perceptions of their daily functions concur, and how other factors may influence both ratings.

**Methods:**

Data of 150 PD patients who underwent cognitive and motor testing, as well as their informants were analyzed. The 10-item Functional Activities Questionnaire (FAQ), completed separately by patients (FAQ-S) and their informants (FAQ-I), assessed ADL function. Weighted κ statistics summarized level of agreement, and a discrepancy score (FAQ-I – FAQ-S) quantified agreement. Correlation analyses between FAQ total scores, patient and informant characteristics, and cognitive scores were conducted, with *post hoc* regressions to determine the associations between both FAQ scores and cognition, independent of patient characteristics.

**Results:**

The sample included 87 patients with normal cognition, 50 with mild cognitive impairment, and 13 with dementia. Overall, there was fair to moderate agreement between patients and informants on individual FAQ items (0.27 ≤ κ ≤ 0.61, *p* < 0.004), with greater discrepancies with increasing cognitive impairment. Patients’ age, motor severity, non-motor burden, and depression also affected both ratings (0.27 ≤ *r* ≤ 0.50, *p* < 0.001), with motor severity showing the greatest influence on both ratings. Both the FAQ-I and FAQ-S were correlated with almost all cognitive domains. *Post hoc* regression analyses controlling for patient characteristics showed that the attention domain was a significant predictor of both the FAQ-S and FAQ-I scores, and memory was also a significant predictor of the FAQ-I score. Only 29.3% of patients agreed perfectly with informants on the FAQ total score, with informants most commonly rating ADL impairments as more severe than patients.

**Conclusions:**

Patient and informant ratings of ADL function using FAQ items showed moderate agreement, with only few items reaching substantial agreement. Ratings of both were associated with patient cognitive status, but also other characteristics. In addition to patient and informant reports, objective measures are needed to accurately classify ADL deficits in PD.

## Introduction

Significant impairments in activities of daily living (ADL) function, in addition to impaired cognition, are the core criterion for diagnosing Parkinson’s Disease (PD) dementia (PDD) (Emre et al., 2007). Both ADL impairments and severe cognitive impairment result in increased risk for nursing home placement and mortality (Hosking et al., 2021). Recent studies have shown that even patients with mild cognitive impairment (PD-MCI) display first signs of ADL dysfunction (Pirogovsky et al., 2014; Cheon et al., 2015; Fellows and Schmitter-Edgecombe, 2019), possibly indicating a group at risk for dementia (Beyle et al., 2018). As the diagnosis of ADL deficits requires these to be solely caused by cognitive deficits, an important step for accurate diagnoses is the measurement of ADL deficits in PD.

Insight into patients’ general ADL function is commonly given by a reliable informant, such as a spouse or close friend (Cahn-Weiner et al., 2007). Research in Alzheimer’s Disease shows that while informant reporting accurately reflects the functional changes, there are variations in the quality of their reports (Farias et al., 2017). Furthermore, caregiver stress and any depressive symptoms of the caregiver have an influence on the external assessment of ADL in mild dementia patients (Zanetti et al., 1999; Razani et al., 2007). Factors such as caregiver age, education level, living situation, and the nature of the relationship to the patient have been reported to influence the caregivers’ assessments in Alzheimer’s Disease (Lin et al., 2017), and also in PD (Bhimani, 2014; Mosley et al., 2017; Kalampokini et al., 2020). Additional negative influences on caregivers of PD patients include cognitive status, disease duration, and patients’ motor symptom severity (Caap-Ahlgren and Dehlin, 2002; Ransmayr, 2020).

Self- reports are also used to gain perspective on how ADL impairments affect the patient’s functioning (Foster and Hershey, 2011). It is important to note that the assessment of ADL impairments in elderly patients becomes more difficult with increasing cognitive deficits; patients with dementia often lack insight to correctly perceive the severity of their illness (Farias et al., 2017). PD patients also tend to underestimate their abilities with increasing cognitive decline (Seltzer et al., 2001), and previous studies have shown PD patients rate themselves as less impaired on measures of ADL than their caregivers (Leritz et al., 2004). Compared to objective measures, 44% of all study participants underestimated their ADL impairment, while 13% overestimated impairment (Shulman et al., 2006). Patients who underestimated ADL disabilities had shorter disease durations, more preserved cognitive abilities, and were living in a family environment, while those who overestimated their ADL skills had advanced PD, showed cognitive dysfunctions, and lived alone (Shulman et al., 2006).

In contrast to the above-mentioned studies, other researchers have not been able to find differences in caregiver versus self-report of ADL disabilities in PD patients (Brown et al., 1989; Liepelt-Scarfone et al., 2013; Copeland et al., 2016). More studies are needed to determine whether self-reports or informant-reports are more useful for judging impairments in ADL function in the clinical routine, as the loss of functional independence is an important outcome of disease progression (Santos Garcia et al., 2021). The aim of this study was therefore to examine both self- and informant-reported ADL using a widely known questionnaire in a cohort of PD patients with varying degrees of cognitive impairment. We aimed to look at the agreement between both sources as well as associations with both patient and informant characteristics, hypothesizing that there would only be moderate agreement between both sources regarding ADL function, with increasing divergence relating to the severity of cognitive impairment.

## Materials and Methods

### Design and Recruitment

Between July 2018 and September 2020, 270 PD patients were recruited to take part in the cross-sectional “Cognitive-driven ADL impairment as a predictor for Parkinson’s disease dementia (PDD)” study. Inclusion criteria included: age between 50 and 90 years of age, diagnosis of PD according to UK Brain Bank Criteria, and the ability to understand study requirements and communicate with investigator. Exclusion criteria included: other neurodegenerative disease interfering with cognition or preventing the ability to give informed consent, alcohol, medication or drug dependency or abuse (except for nicotine), or participation in a clinical investigation of a new compound within the last 4 weeks. Additionally, all patients were asked to designate one person to be their informant who was then contacted to give information regarding the patient. This study was approved by the Ethics Committee of the Medical Faculty of the University of Tübingen (284/2018BO1). All patients (or their proxies if necessary) and their informants provided written, informed consent.

Of all patients invited to participate, 36 (13.3%) met exclusion criteria and 52 (19.3%) declined to take part in the examination. A total of 182 (67.4%) patients were included in the study and assessed. For the following final analyses, 17 (9.3%) patients who had received deep brain stimulation (DBS) implantation prior to assessment were excluded. We chose to exclude these patients as it is currently unclear how DBS affects ADL: some studies have shown improvements on ADL functions (Gorecka-Mazur et al., 2019; Cernera et al., 2020), while others demonstrated no effect on ADL function (Pusswald et al., 2019). Two patients (1.1%) were additionally excluded as medication intake interfered with correct classification of cognitive status. Thirteen (7.1%) patients had missing FAQ data (*n* = 9 did not have an informant and *n* = 4 unable to fill out the FAQ themselves due to severely impaired cognition) and were also excluded. In total, data of 150 patients (and their informants) was analyzed in the final dataset.

### Assessments

The Functional Activities Questionnaire (FAQ) (Pfeffer et al., 1982) was used to assess ADL impairments. It consists of 10 items assessing instrumental ADL functions, where capability of each item is rated from 0 (normal) to 3 (dependent on others). A maximum score of 30 points can be achieved, and higher scores indicate greater severity of ADL impairment. The FAQ was separately administered to both the patient as a self-report (FAQ-S) and the informant (FAQ-I) to evaluate the patients’ ADL functioning in the previous 4 weeks.

#### Patient Measures

Patient demographics (age, sex, education years) and medical history [age at onset of PD, disease duration, and anti-parkinsonian medication intake expressed using the levodopa equivalent daily dose (LEDD) (Tomlinson et al., 2010)] were collected. Motor function was assessed by a trained specialist using the Movement Disorder Society Unified Parkinson’s Disease Rating Scale Part III (MDS-UPDRS-III) (Goetz et al., 2008). Depression was assessed using the Beck Depression Inventory-II (BDI-II) (Beck et al., 1996), a 21-item instrument quantifying depressive symptoms over the last 2 weeks. Non-motor symptom burden was assessed using the Parkinson’s Disease Non-Motor Symptoms Questionnaire (NMSQ) (Chaudhuri et al., 2006).

Global cognitive functioning was measured using the Montreal Cognitive Assessment (MoCA) (Nasreddine et al., 2005). A comprehensive neuropsychological battery assessing five cognitive domains was administered:

- *Attention*: Letter-Number-Sequencing and Digit-Symbol subtests of the Wechsler Intelligenztest für Erwachsene [WIE, German adaptation of the Wechsler Intelligence Test for Adults, (Aster et al., 2006)];
- *Executive Functions*: Trail Making Test-Part B, semantic fluency, and phonemic fluency subtests from the Consortium to Establish a Registry for Alzheimer’s Disease–Plus Battery (CERAD-PLUS) (Morris et al., 1988);
- *Memory*: Word List learning, Word List recall, Word List discriminability, and Constructional Praxis recall subtests of the CERAD-PLUS;
- *Visuospatial Functions*: Constructional Praxis subtest of the CERAD-PLUS and Fragmented Words subtest of the “Leistungsprüfsystem 50 + “ [performance test for older adults 50–90 years, (Sturm et al., 1993)];
- *Language*: Similarities subtest from the WIE and the modified Boston Naming Test of the CERAD-PLUS.

All raw scores were converted to *z*-scores using test manuals, adjusting for age and/or education where appropriate, and composite domain *z*-scores were calculated for each cognitive domain. Cognitive tests assigned to each domain were chosen according to the recommendations of the Movement Disorders Task Force (Litvan et al., 2012) and this comprehensive battery been previously used in a study with PD patients (Becker et al., 2020). The CERAD-PLUS battery has been shown to be accurate and useful in identifying cognitive impairment in PD patients (Karrasch et al., 2013; Camargo et al., 2018), while both the WIE [English version: Wechsler Adult Intelligence Scale (Yamawaki et al., 2020; Chen et al., 2021)] and the Leistungsprüfsystem 50 + batteries have been utilized in PD-cognition studies (Fengler et al., 2016; Kalbe et al., 2016). Furthermore, a recent systematic literature review identified specific tests used in PD research that have been normed for German-speaking populations and their corresponding cognitive domain (Liepelt-Scarfone et al., 2021). The authors presented guidelines for the neuropsychological assessment of PD patients in the German language, with their findings supporting our chosen tests and domains.

Patients were classified as cognitively normal (PD-CN) if no cognitive or ADL impairment was present. PD-MCI was diagnosed according to the Level-II criteria of the Movement Disorders Task Force (Litvan et al., 2012) if impairment (1.5 standard deviations below population norms) was present in at least two cognitive tests, with preserved ADL functioning. PDD was diagnosed according to consensus criteria (Dubois et al., 2007) if both cognitive impairment and severe impairments in ADL function were present. Fourteen patients (*n* = 1 PD-CN, *n* = 4 PD-MCI, and *n* = 9 PDD) were unable to complete the neuropsychological test battery for various reasons (e.g., severe cognitive impairment, physical and/or mental exhaustion especially toward the end of the test battery). Cognitive diagnoses for these patients were made according to available cognitive data (*z*-scores of the tests the patients did complete), agreements between informants and clinicians regarding ADL status, and neuropsychological investigator judgment. Available medical data (e.g., if the patient had received a diagnosis of either PD-MCI or PDD from a neuropsychologist, neurologist, or primary physician prior to examination) and previous cognitive evaluations were also taken into consideration.

#### Informant Measures

Demographic information was collected from each informant, including age, sex, education years, living situation, and how many times per week (1× a week, 2–3× per week, or daily) they saw the patient. The Bayer-ADL scale (Hindmarch et al., 1998) was given to informants to assess instrumental ADL, where the patient’s ability to perform 25 tasks is rated from 0 “never” to 10 “always.” The total sum score is divided by the number of questions answered to obtain a scaled score, ranging from 1 to 10, where higher values indicate more severe impairments in ADL function. Scoring is as follows: 1.0–2.0, no difficulties with ADL, 2.1–5.0 mild difficulties with everyday function, and 5.1–10.0 indicates clear difficulties in coping with everyday life.

### Statistical Analyses

REDCap electronic data capture tools (Harris et al., 2009) hosted at the Hertie Institute for Clinical Brain Research was used to collect and manage study data. SPSS Version 27 (IBM Corp., Armonk, NY, United States) was used to conduct all statistical analyses, with α levels set at 0.05. For missing demographic patient data [*n* = 12 (8%) MDS-UPDRS-III, *n* = 4 (2.7%) NMSQ, and *n* = 2 (1.3%) BDI-II], median values per cognitive group status were imputed to compensate for any missing values. Analyses involving cognitive data only included only those patients who had completed all tests (*n* = 136) to ensure equal representation of each averaged domain *z*-score. The Shapiro-Wilk tested assumptions of normality. As data were not normally distributed, demographic variables were examined using the non-parametric Pearson Chi-square, Mann-Whitney *U* tests, or Independent-Samples Jonckheere-Terpstra Tests for Ordered Alternatives where appropriate.

Weighted κ statistics with linear weights were used to summarize the level of agreement between patient and informants for each individual FAQ item. Agreement values were interpreted as follows: 0–0.20 slight, 0.21–0.40 fair, 0.41–0.60 moderate, 0.61–0.80 substantial, and 0.81–1.00 almost perfect (Landis and Koch, 1977). The FAQ total score was compared between raters using Intraclass Correlation (ICC) using an absolute-agreement, two-way mixed effects model based on single measurements. To determine how different factors may influence the level of agreement, analyses were re-run using the following stratifications: (i) cognitive status of the patient [non-demented (PD-CN and PD-MCI) and demented (PDD)], (ii) sex of the patient, (iii) disease duration using a median split (duration ≤ 7.31 years and duration > 7.32 years), and (iv) BDI-II using a median split (score ≤ 9 and score > 10).

Spearman’s rank correlations between the total FAQ-S and FAQ-I scores, cognitive scores, patient demographic variables of interest (age, sex, education, disease duration, UPDRS-III total score, BDI-II score, and NMSQ score), caregiver variables of interest (age, education years), and the Bayer-ADL were conducted. *Post hoc* multivariate linear regressions were conducted to determine the associations between both FAQ scores and cognition, independent of patient characteristics. Ten regressions were run for each of the five cognitive domains separately (due to multicollinearity when all domains are added into one model), with both the FAQ-S and the FAQ-I as the dependent variable. Further covariates in the models included patient age, UPDRS-III score, NMSQ scores and BDI-II score, as these were all significantly correlated with both FAQ scores. For these regressions, the α was adjusted to 0.005 to correct for multiple comparisons (0.05/10).

Lastly, a discrepancy score (D) was calculated for the total FAQ score between informant and self-ratings: FAQ-I – FAQ-S. Positive values denoted higher impairment rated by informants (D_I_), negative values denoted higher impairment rated by patients themselves (D_S_), and scores of 0 indicated perfect agreement between informants and patients (D_A_). Patients were split according to the discrepancy score, and demographic and cognitive variables were compared between groups using independent samples Kruskal-Wallis *H* tests, with *post hoc* Bonferroni corrections for multiple testing.

## Results

Of all 150 PD patients, 87 (58%) were classified as PD-CN, 50 (33.3%) as PD-MCI, and 13 (8.7%) as PDD. The Jonckheere-Terpstra test showed a significant effect of education between groups, and *post hoc* analyses with Bonferroni correction for multiple tests revealed PD-MCI patients had significantly lower years of education than PD-CN patients (*p* = 0.03; see Table 1 for details). Significant differences were found between all three groups for the UPDRS-III total score, where PDD patients had the most severe motor impairment according to *post hoc* analyses (PD-CN < PD-MCI < PDD, *p* < 0.002). Analyses also showed PDD patients also had a significantly higher non-motor symptom burden than PD-CN patients (*post hoc p* = 0.03). The BDI-II was statistically different between groups, however, *post hoc* significances did not reveal any specific group differences after correction for multiple testing. For measures of ADL, both the FAQ-I and FAQ-S total scores as well as the Bayer-ADL score were significantly different between groups, with PDD patients again showing the most severe impairments in ADL (*post hoc* PD-CN < PD-MCI < PDD, *p* < 0.001).

**TABLE 1:** Patient characteristics according to cognitive status.

	PD-CN *n* = 87	PD-MCI *n* = 50	PDD *n* = 13	*p*-value
***Demographics***				
Male sex: *n* (%)	58 (66.7)	27 (54)	9 (69.2)	0.30
Age (y)	68.45 (51.99–83.47)	68.13 (52.97–83.67)	73.35 (67.69–82.30)	0.17
Education (y)	13 (8–21)	12 (8–19)	12 (11–18)	0.02*
Age at onset (y)	60.48 (39–75.54)	58.77 (44.75–76.58)	64.88 (54.17–76.65)	0.45
Disease duration (y)	7.07 (3.28–21.74)	8.05 (2.15–20.22)	7.42 (5.57–13.92)	0.36
UPDRS-III	22.5 (3–50)	30 (8–68)	45 (27–56)	<0.001**
LEDD	700 (100–1950)	702 (52–1510)	540 (285–1050)	0.45
NMSQ	8 (1–22)	9.5 (0–24)	11 (8–23)	0.03*
BDI-II	8 (0–35)	10 (0–33)	12 (4–27)	0.02*
***ADL***				
FAQ-I total score	1 (0–24)	3.50 (0–24)	19 (7–28)	<0.001**
FAQ-S total score	0 (0–22)	1 (0–24)	12 (1–28)	<0.001**
Bayer-ADL	1.42 (1–6.12)	2.32 (1–7.20)	6.33 (2.20–9.56)	<0.001**
***Cognition‡***				<0.001**
MoCA total score	27 (19–30)	24.5 (17–30)	18 (17–19)	<0.001**
Attention	0.20 (−1.20–1.90)	−0.45 (−2.60–1.20)	−1.10 (−2.10−0.70)	<0.001**
Executive functions	0.27 (−2.00–2.57)	−0.83 (–1.93–1.27)	−1.15 (–1.83– −0.70)	<0.001**
Memory	0.29 (−1.90–1.30)	−1.05 (−2.95–0.38)	−1.87 (−2.90– −0.77)	<0.001**
Visuospatial functions	−0.15 (−1.70–1.45)	−1.20 (−2.50–1.00)	−1.62 (−2.65– −1.30)	<0.001**
Language	0.43 (−0.70–1.40)	−0.30 (−1.85–1.35)	−0.47 (−0.70– −0.05)	<0.001**

Results are given as Median (Range) unless otherwise indicated, *p < 0.05, **p < 0.01. ‡Due to missing values, cognitive domain scores only computed and analyses run for 136 patients. ADL, activities of daily living; BDI-II, Beck Depression Inventory-II; FAQ-I, Functional Activities Questionnaire Informant-rated; FAQ-S, Functional Activities Questionnaire Self-rated; H&Y, Hoehn and Yahr; LEDD, levodopa equivalent daily dose; MoCA, Montreal Cognitive Assessment; NMSQ, Non-Motor Symptoms Questionnaire; PD-CN, Parkinson’s Disease cognitively normal; PDD, Parkinson’s Disease dementia; PD-MCI, Parkinson’s Disease with mild cognitive impairment; UPDRS-III, Unified Parkinson’s Disease Rating Scale Part III; y, year.

Comparisons of cognitive data were done using data of 136 patients with complete neuropsychological testing (PD-CN *n* = 86, PD-MCI *n* = 46, PDD *n* = 4). Global cognition measured using the MoCA showed significant differences between groups, with PDD patients exhibiting the most impaired cognitive performance (*post hoc* PD-CN > PD-MCI > PDD, *p* < 0.008). All five cognitive domains were significantly different between groups, following the same *post hoc* pattern where PD-MCI and PDD patients performed similarly, and PD-CN patients performed the best (PD-CN > PD-MCI = PDD, *p* < 0.005).

Regarding informants, they were most frequently spouses (118, 78.7%), followed by children/stepchildren (13, 8.7%), life partner (8, 5.3%), close friend (6, 4%), other relative (3, 2%), and siblings (2, 1.3%) of the patients. Informants were also predominantly females (101, 67.3%), with a median age 64 (range 29–86) and median of 13 years total education (range 8–28). Regarding time spent with the patient, 121 (87.7%) informants reported seeing them daily, 10 (7.2%) two to three times a week, and 7 (5.1%) only once a week.

### Agreement Statistics

Table 2 shows the weighted κ statistics for each FAQ item when examining the entire sample. Item 10 (traveling out of house) was rated as substantial agreement (κ = 0.61, *p* < 0.01), with most other items reaching moderate or fair agreement (0.27 ≤ κ ≤ 0.59, *p* < 0.004). A good degree of reliability was found between patient and informant total scores on the FAQ. The single measure ICC was 0.73 with a 95% confidence interval 0.61–0.81 [*F*(149,149) = 7.24, *p* < 0.001]. Splitting patients according to cognitive status revealed that non-demented (PD-CN and PD-MCI) patients and their informants showed fair to moderate agreement on all items (0.24 ≤ κ ≤ 0.57, *p* < 0.004), but only 3 items (1, 5, and 10) actually reached the moderate level (see Table 3). Agreement between demented patients and their informants was due to chance for almost all items (0.08 ≤ κ ≤ 0.38, *p* > 0.09). Only the FAQ items 3 (shopping alone) and 8 (paying attention) demonstrated moderate agreement (κ = 0.56 and κ = 0.51, respectively; *p* < 0.01) between patient and informant ratings.

**TABLE 2:** Differences between self and informant ratings of the FAQ.

FAQ item	Self	Informant	Weighted κ	SE of κ	*p*-value
1. Handling finances	20 (13.3)	36 (24)	0.59	0.08	<0.001**
2. Assembling tax records	47 (31.3)	56 (37.3)	0.47	0.07	<0.001**
3. Shopping	27 (18)	41 (27.3)	0.48	0.08	<0.001**
4. Skills and hobbies	21 (14)	58 (38.7)	0.27	0.06	<0.001**
5. Using appliances	12 (8)	24 (16)	0.48	0.11	<0.001**
6. Meal preparation	31 (20.7)	52 (34.7)	0.37	0.08	<0.001**
7. Current events	13 (8.7)	26 (17.3)	0.37	0.10	<0.001**
8. Paying attention	20 (13.3)	30 (20)	0.40	0.08	<0.001**
9. Remembering appointments	37 (24.7)	48 (32)	0.39	0.07	<0.001**
10. Traveling out of house	26 (17.3)	42 (28)	0.61	0.07	<0.001**

Results are expressed as Number of patients scoring ≥ 1 (%), **p < 0.01. FAQ, Functional Activities Questionnaire; SE, standard error.

**TABLE 3:** Agreement between self and informant ratings of the FAQ according to cognitive status.

	Non-demented PD patients *n* = *137*					Demented PD patients *n* = *13*				
FAQ item	Self	Informant	Weighted κ	SE of κ	*p*-value	Self	Informant	Weighted κ	SE of κ	*p*-value
1. Handling finances	11 (8)	23 (16.8)	0.51	0.11	<0.001**	9 (69.2)	13 (100)	0.17	0.12	0.18
2. Assembling tax records	37 (27)	43 (31.4)	0.37	0.08	<0.001**	10 (76.9)	13 (100)	0.08	0.07	0.26
3. Shopping	19 (13.9)	30 (21.9)	0.38	0.09	<0.001**	8 (61.5)	11 (84.6)	0.56	0.17	0.002**
4. Skills and hobbies	14 (10.2)	50 (36.5)	0.24	0.07	<0.001**	7 (53.8)	8 (61.5)	0.13	0.18	0.52
5. Using appliances	7 (5.1)	15 (10.9)	0.45	0.15	<0.001**	5 (38.5)	9 (69.2)	0.29	0.20	0.15
6. Meal preparation	24 (17.5)	41 (29.9)	0.29	0.08	<0.001**	7 (53.8)	11 (84.6)	0.21	0.19	0.28
7. Current events	10 (7.3)	18 (13.1)	0.39	0.11	<0.001**	3 (23.1)	8 (61.5)	0.18	0.22	0.33
8. Paying attention	15 (10.9)	24 (17.5)	0.33	0.09	<0.001**	5 (38.5)	6 (46.2)	0.51	0.14	0.01*
9. Remembering appointments	26 (19)	37 (27)	0.30	0.09	<0.001**	11 (84.6)	11 (84.6)	0.20	0.20	0.27
10. Traveling out of house	19 (13.9)	32 (23.4)	0.57	0.09	<0.001**	7 (53.8)	10 (76.9)	0.38	0.19	0.09

Results are expressed as Number of patients scoring ≥ 1 (%), *p < 0.05, **p < 0.01. FAQ, Functional Activities Questionnaire; PD, Parkinson’s Disease; SE, standard error.

Next, the role of sex of the patient was examined. There was a slight tendency for better agreements when the patient was a male than if they were females (Supplementary Table 1). The agreement of ratings for male patients ranged from fair to substantial (0.30 ≤ κ ≤ 0.68, *p* < 0.002), while ratings for female patients were fair to moderate (0.23 ≤ κ ≤ 0.55, *p* < 0.008) and one item with a slight agreement due to chance (κ = 0.10, *p* = 0.30). Patients were then split according to median years of disease duration (Supplementary Table 2). For patients with a shorter disease duration, item 5 (using appliances) was rated as substantial (κ = 0.62, *p* < 0.001), with all other items reaching a fair or moderate agreement (0.22 ≤ κ ≤ 0.53, *p* < 0.01). The group of patients with longer disease duration also showed fair to moderate agreement (0.24 ≤ κ ≤ 0.46, *p* < 0.01), and items 1 (handling finances) and 10 (traveling out of house) reached substantial agreement (κ = 0.62 and κ = 0.69, respectively; *p* < 0.001). Lastly, patients were split according to median BDI-II score (Supplementary Table 3). Those patients with lower depressive symptomatology demonstrated substantial agreement on three items (0.62 ≤ κ ≤ 0.72, *p* < 0.001), while the others showed fair or moderate agreement (0.31 ≤ κ ≤ 0.52, *p* < 0.001). Patients with higher depressive symptoms showed overall less agreement than those with higher symptoms. Almost all items had fair to moderate agreement (0.32 ≤ κ ≤ 0.59, *p* < 0.01), while item 4 (skills and hobbies) had only slight agreement (κ = 0.17, *p* = 0.02).

For all agreement analyses run, the number of ratings of items ≥ 1 (indicating at least mild difficulties with the daily task) was consistently higher for informant ratings than for the self-ratings. We examined the consistency of the individual item agreement statistics *post hoc*, to determine whether some FAQ items consistently showed better or worse agreement than others. This was done by ranking the weighted κ statistics of all analyses according to FAQ item and examining the frequency of the items corresponding to the top and bottom three ranks. There was a clear tendency for the items 10 (traveling out of house), 1 (handling finances and balancing checkbook), and a tie between 3 (shopping alone) and 5 (using household appliances) to show the most agreement (top ranked in 8, 7, 4, and 4 analyses, respectively). The items 4 (engaging in skills and hobbies), 6 (preparing a balanced meal), and 7 (keeping up with current events) were those with the poorest consistent agreement (bottom ranked in 8, 6, and 6 analyses, respectively).

### Correlation Analyses

The FAQ-S and FAQ-I total scores were moderately positively correlated with one another (*r*_*s*_ = 0.61, *p* < 0.001). Table 4 shows the correlations for the FAQ scores and both patient and caregiver variables of interest. Both the FAQ-S and FAQ-I total scores were positively correlated with patients’ age, motor severity, number of non-motor symptoms, and depressive symptoms (0.27 ≤ *r*_*s*_ ≤ 0.50, *p* < 0.001), and negatively correlated with patients’ education (*r*_*s*_ = −0.21 and *r*_*s*_ = −0.18, respectively; *p* < 0.03). The Bayer-ADL score was strongly positively correlated with the FAQ-I (*r*_*s*_ = 0.85, *p* < 0.001) and moderately correlated with the FAQ-S (*r*_*s*_ = 0.55, *p* < 0.001). Examining the cognitive data, both the FAQ-S and FAQ-I scores were negatively associated with the MoCA (*r*_*s*_ = −0.33 and *r*_*s*_ = −0.38, respectively; *p* < 0.001). The FAQ-S was significantly negatively correlated with the attention, executive functions, memory, and visuospatial functions domains (−0.40 ≤ *r*_*s*_ ≤ −0.19, *p* < 0.02), but not with language. In contrast, for the FAQ-I, there was a significant negative correlation with all cognitive domains (−0.43 ≤ *r*_*s*_ ≤ −0.21, *p* < 0.02).

**TABLE 4:** Correlations between the total FAQ-S and FAQ-I scores and both patient and caregiver variables of interest.

	FAQ-S	FAQ-I
***Patient characteristics***		
Patient age	0.27**	0.34**
Patient education	−0.21**	−0.18*
Disease duration	0.05	0.13
UPDRS-III	0.38**	0.50**
NMSQ	0.35**	0.36**
BDI-II	0.27**	0.35**
***Patient cognition‡***		
MoCA total score	−0.33**	−0.38**
Attention	−0.38**	−0.43**
Executive functions	−0.31**	−0.36**
Memory	−0.40**	−0.31**
Visuospatial functions	−0.19*	−0.21*
Language	–0.14	−0.21*
***Caregiver characteristics***		
Caregiver age	0.06	0.11
Caregiver education	−0.18*	–0.14
Bayer-ADL	0.55**	0.85**

*p < 0.05, **p < 0.01. ‡Due to missing values, cognitive domain scores only computed and analyses run for 136 patients. ADL, activities of daily living; BDI-II, Beck Depression Inventory-II; FAQ-I, Functional Activities Questionnaire Informant-rated; FAQ-S, Functional Activities Questionnaire Self-rated; MoCA, Montreal Cognitive Assessment; NMSQ, Non-Motor Symptoms Questionnaire; UPDRS-III, Unified Parkinson’s Disease Rating Scale Part III.

### *Post hoc* Regression Analyses

All ten linear regression models with either the FAQ-S or FAQ-I as the dependent variable were stable. The linear regression with attention as the cognitive independent and FAQ-S as the dependent variable was statistically significant [*F*(5,130) = 8.27, *p* < 0.001], explaining 24.1% (Nagelkerke *R*^2^) of the variance. Attention significantly predicted the FAQ-S (unstandardized β = −1.49, standard error of β = 0.46, *p* = 0.001), as did the UPDRS-III score (unstandardized β = 0.09, standard error of β = 0.03, *p* = 0.004). The model including memory was also significant [*F*(5,130) = 7.50, *p* < 0.001], explaining 22.4% (Nagelkerke *R*^2^) of the variance. Memory domain as a predictor did not reach clinical significance after correction for multiple testing (unstandardized β = −0.99, standard error of β = 0.36, *p* = 0.007), while the UPDRS-III score was again a significant predictor (unstandardized β = 0.11, standard error of β = 0.04, *p* = 0.001). For the FAQ-S models including executive functions, visuospatial functions, and language domains, only the UPDRS-III score was a significant predictor (*p* < 0.004).

When the dependent variable was the FAQ-I, the model including attention was again significant [*F*(5,130) = 10.48, *p* < 0.001], explaining 28.7% (Nagelkerke *R*^2^) of the variance. Attention significantly predicted the FAQ-I (unstandardized β = −1.75, standard error of β = 0.56, *p* = 0.002), as did the UPDRS-III score (unstandardized β = 0.11, standard error of β = 0.04, *p* = 0.005) and patient age (unstandardized β = 0.18, standard error of β = 0.05, *p* < 0.001). When memory was the independent variable, the model was again significant [*F*(5,130) = 10.12, *p* < 0.001], explaining 28% (Nagelkerke *R*^2^) of the variance. Significant predictors of the FAQ-I were memory (unstandardized β = −1.27, standard error of β = 0.44, *p* = 0.004), UPDRS-III score (unstandardized β = 0.12, standard error of β = 0.04, *p* = 0.002), and patient age (unstandardized β = 0.19, standard error of β = 0.05, *p* < 0.001). The FAQ-S models including executive functions, visuospatial functions, and language domains, were significant, however, the only significant predictors were patient age (*p* < 0.001) and UPDRS-III score (*p* < 0.001).

### Discrepancy Scores

The discrepancy score based on difference between the FAQ-S and FAQ-I total scores showed that 76 (50.7%) of informants rated ADL function as more impaired than their patients (D_I_ group), 44 (29.3%) of participants agreed with their informants (D_A_ group), and 30 (20%) patients rated their impairment worse than informants (D_S_ group). Cognitive group distribution was as follows: D_I_ – 38 PD-CN, 27 PD-MCI, 11 PDD; D_A_ – 34 PD-CN, 10 PD-MCI; D_S_ – 15 PD-CN, 13 PD-MCI, 2 PDD.

Kruskal-Wallis *H* tests showed group differences for patient age [*H*(2) = 6.59, *p* = 0.04], and *post hoc* Bonferroni corrected analyses revealed patients in the D_I_ group were significantly older than patients in the D_A_ group (*p* = 0.04). A significant effect was found for the MDS-UPDRS-III score [*H*(2) = 19.29, *p* < 0.001], where motor severity was greater in the D_I_ group than in the D_A_ group (*p* < 0.001). The NMSQ was also significantly different between groups [*H*(2) = 11.94, *p* = 0.003], with patients in the D_A_ group reporting less non-motor symptoms than both the D_S_ (*p* = 0.03) and D_I_ (*p* = 0.003) groups. Depressive symptomatology was also different between groups [*H*(2) = 11.29, *p* = 0.004], where patients in the D_I_ group had higher BDI scores than those in the D_A_ group. The Bayer ADL scale score was significantly different between groups, [*H*(2) = 57.82, *p* < 0.001], with *post hoc* analyses showing D_A_ < D_S_ < D_I_, *p* < 0.02 for all. Neither caregiver age [*H*(2) = 0.66, *p* = 0.72] nor education level [*H*(2) = 2.15, *p* = 0.34] were significantly different between groups.

**FIGURE 1:**
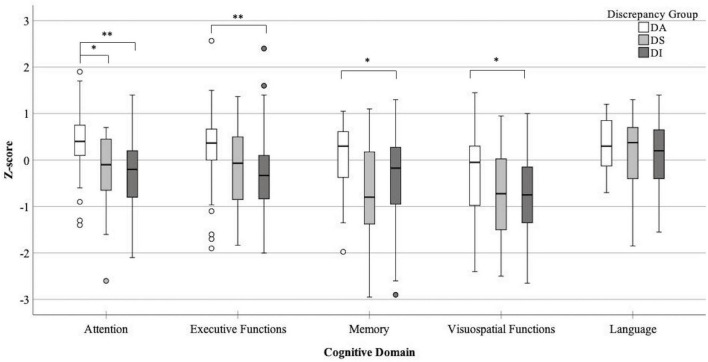
Box-and-whisker plot detailing performance across all five cognitive domains for each discrepancy score group (DA- perfect agreement between informants and patients, DS- higher impairment rated by patients, DI- higher impairment rated by informants), *p < 0.05, ^**^p < 0.001.

For the cognitive data, a significant effect for the MoCA was found [*H*(2) = 9.47, *p* = 0.009], where patients in the DI group had significantly lowered global cognition than patients in the DA group (*p* = 0.006). The attention domain showed a significant group effect [*H*(2) = 22.49, *p* < 0.001] where patients in the D_A_ had significantly better cognition than patients in both the D_S_ and D_I_ groups (*p* < 0.01). Significant group effects were also found for the executive functions [*H*(2) = 13.45, *p* = 0.001], memory [*H*(2) = 9.85, *p* = 0.007], and visuospatial functions [*H*(2) = 9.68, *p* = 0.008] domains. *Post hoc* results showed patients in the D_A_ had significantly better cognition than patients in the D_I_ groups (*p* < 0.01). For the visuospatial domain, patients in the D_A_ had borderline insignificant (*p* = 0.05) better cognitive scores than patients in the D_S_ group. No effect was found between groups for performance in the language domain [*H*(2) = 1.62, *p* = 0.45]. Figure 1 shows a box-and-whisker plot of performance on each cognitive domain across discrepancy score groups including *post hoc* significant differences.

## Discussion

This study aimed to examine the agreement between self- and informant-reports of ADL function and determine whether they were affected by different patient or informant characteristics. The main results were that: (i) agreement analyses showed overall fair to moderate agreement, with few items reaching substantial agreement in various sub-analyses; (ii) both patient and informant ratings were significantly correlated with patient characteristics including cognition, as well as motor severity, age, and depression; and (iii) informants most commonly rated ADL impairments as more severe than patients, with only 29.3% of patients showing perfect agreement with informants on the their ADL performance as reflected by the FAQ total score.

Severe deficits in daily functioning are the core criteria for diagnosing PDD, however, studies have shown that even PD-MCI patients can show early, non-clinical signs of ADL dysfunction (Pirogovsky et al., 2014; Beyle et al., 2018). It is important to understand how and when changes in ADL indicative of dementia in PD emerge to be able to provide early interventions for maintaining patients’ autonomy and, as a direct consequence, their quality of life. However, to be able to recognize significant changes in ADL indicating first signs of PDD, reliable and valid assessments with high diagnostic accuracy are needed. Our current results showed that when using the FAQ, there was only a fair to moderate agreement between patients and their informants regarding the daily functional abilities of the patient. The data also support previous findings that there was some level of over- and underreporting done by either patients or caregivers (Shulman et al., 2006; Christ et al., 2013; Cholerton et al., 2020), which is important to take into account when using these questionnaires to screen for ADL impairment indicative of dementia. To the authors knowledge, only one other study has directly examined inter-rater agreement of ADL ratings by giving both patients and informants the same scale (Deck et al., 2019). Patient and caregiver agreement was examined with the Penn Parkinson’s Daily Activities Questionnaire (PDAQ-15), a scale designed to assess instrumental cognitive-associated ADL impairment in PD, and additionally compared to an objective functional measure of ADL. The authors of this study found a moderate agreement between patient and informant ratings on the total (ICC = 0.57) and individual items of the PDAQ-15 (Deck et al., 2019), similar to our current findings reported for the FAQ. Together these results show that agreement between patients and informants is not perfect, regardless of the scale used and the underlying constructs measured. It is imperative to for clinicians and researchers alike to consider that using a single measure of ADL dysfunction, whether rated by the patient or informant, to detect and diagnose presence of PDD potentially overestimates the motor effects on ADL function as well as underestimating cognitive sources. Incorporating objective measures of functional performance as well as self or informant-reported questionnaires may aid in determining level of ADL functioning especially in PD-MCI patients who would be able to undergo rigorous testing. Alternatively, consensus rating between both patient and informants should be considered, although studies are needed to determine whether these will better correspond to patient’s real-world abilities than individual assessments.

A specific item analysis was undertaken which showed that traveling out of house, handling finances, going shopping alone, and using household appliances had the highest agreement in our sample, while engaging in skills and hobbies, preparing a balanced meal, and keeping up with current events had the poorest agreement. A previous study showed the specific ADL items managing finances, keeping their appointments, following current events, and using a phone were unaffected by motor symptoms and able to identify dementia in PD patients (Cheon et al., 2015). Furthermore, differentiating cognitive from motor influences on the FAQ showed that both handling finances and keeping up with current events were predicted by cognitive, not motor, abilities (Becker et al., 2020). However, only finances showed adequate agreement within our sample, whereas the agreements between patients and informants regarding following current events were consistently poor. It is possible that impairments in handling finances can be better acknowledged by patients because there is a tangible result which may be cause for concern when impairments are noted, while keeping up with current events is a more subjective experience. Patients may tend to overestimate this ability as they lack a concrete way to measure its loss. On the other hand, traveling out of the house, going shopping alone, and using appliances were all related to motor ADL – as these are affected by PD motor symptoms (e.g., tremor or dyskinesias), it is possible that these are more often noticed by patients and informants alike. This is also supported by the fact that motor severity was a significant factor in predicting both FAQ-S and FAQ-I scores. Patients may be more willing to admit their difficulties in these areas as opposed to, for example, engaging in their skills and hobbies which they are more hesitant to give up due to motor symptoms. Future studies should examine in further detail whether there are specific ADL abilities that may be more prone to disagreements and how both patient and informant perceptions of these abilities can change over time. It is possible that either patient or informant ratings on certain ADL questionnaire items correspond accurately to patients’ real-life functioning. Such analyses can inform judgments regarding severity of ADL deficits as more weight can be given to items and ratings known to reflect patients’ daily life.

Generally, studies have focused on comparing informant-rated and patient-rated ADL functioning to the objective cognitive performance of the patient (Shulman et al., 2006; Copeland et al., 2016; Cholerton et al., 2020). While both patients and their informants may not accurately identify specific cognitive deficits, these studies generally show that in early stages of cognitive decline, patient-ratings may be more sensitive to changes in ADL affected by cognition than those of their informants. Only with increasing cognitive decline does the participant lose awareness of their ADL abilities and the knowledgeable informant report become more valuable. Current results confirmed this previous research that PD patients underestimate their abilities with increasing cognitive decline by rating themselves as less impaired than their informants (Seltzer et al., 2001; Leritz et al., 2004; Shulman et al., 2006; Deck et al., 2019). There was poor agreement which was due to chance for almost all individual FAQ items in PDD patients, indicating that demented patients and their informants cannot agree on the patients’ ability to carry out ADL. Due to the small sample size in our study, however, data needed to be interpreted with caution and validated in larger samples. When examining non-demented patients, agreement ratings tended to be worse than when examining the entire sample. This is interesting, as anosognosia is not necessarily found in earlier stages of cognitive decline, although reports are varied (Pennington et al., 2021). We also examined associations directly with cognitive domains. Both patient and informant ratings were correlated with almost all cognitive domains, showing that ADL impairments present in PD are again associated with cognitive decline. To determine whether and to what extent this effect was influenced by patient characteristics, we conducted additional regression analyses. These showed that both patient and informant FAQ ratings were predicted by patients’ performance on attention and memory domains. Previously, it has been shown that deficits in attention predicted ADL performance in PD, after controlling for confounders including age, sex, and motor impairment (Bronnick et al., 2006; Becker et al., 2020). Our results that attentional deficits, independent of motor or non-motor symptoms, increased ratings of ADL impairment corroborate previous results and highlight that attentional deficits may decline in parallel with ADL deficits. Studies have also demonstrated relationships between memory performance and ADL impairments (Beyle et al., 2018; Foster and Doty, 2021). Future research should confirm how memory and attention deficits impair patients’ ADL function, which may lead to more person-centered interventions and assessment strategies.

Apart from cognition, both patient and informant ratings were influenced by patient characteristics, evidenced by both correlation and agreement analyses performed. Severity of PD symptoms, presence of depression, and disease-related motor complications in quality of life are associated with self-assessment of ADL function (Hobson et al., 2001). Our findings replicate most of these findings and also demonstrate they are associated with informant assessments, by showing that both the FAQ-I and FAQ-S were correlated with patients’ age, motor severity, non-motor symptom burden, and depression symptoms. This is important as more than 90% of PD patients present with at least one NMS (Barone et al., 2009) and around 35% present with clinically significant depressive symptoms (Reijnders et al., 2008). Clinicians evaluating ADL deficits should ask whether and to what extent these symptoms are affecting daily functioning, to ascertain a more reliable index of ADL function. Perhaps the most important finding is that patients’ motor severity influences all ADL ratings, and even modulates the association between cognition and ADL ratings. Previous studies have shown that motor symptoms of PD affect ADL (Martinez-Martin et al., 2003; Skinner et al., 2015) and are independent of the cognitive-driven ADL aspects (Becker et al., 2020). However, the diagnosis of PDD requires ADL deficits to be caused by cognitive and not motor problems; as both patient and informant reports are influenced by motor severity, this further emphasizes the need for accurate ADL scales that can capture deficits independent of motor influences. Furthermore, we found an effect of the patients’ sex, where there was a slight tendency for better agreement for male than female patients. This is in line with a previous study that found that women with PD were likely to report more severe ADL dysfunction (Medijainen et al., 2015). This is an interesting finding that should be replicated in future studies, as it would be crucial for clinicians to consider sex differences when judging ADL deficits. Notably, neither informant nor self-ratings were influenced by caregiver age or education, which is not in line with previous literature (Bhimani, 2014; Kalampokini et al., 2020). However, we did not evaluate more specific informant details, such as caregiver burden, depression, or social life, all of which have been shown to have an effect on the reporting of ADL function. Future studies are needed to determine more specifically how and to what extent these caregiver attributes affect their ratings, and whether there are ways to partialize these effects out.

Lastly, discrepancy scores were used to quantify the agreement between patients and informants. Generally, informants rated the ADL impairments as more severe than the patients did, and only 30% of patients and informants agreed regarding the total score. Notably, there were no PDD patients where there was an agreement between both raters, further confirming that PD patients show worse insight into their ADL deficits with worsening cognition. We found that the group where informants rated ADL deficits as more severe were older, had worse motor severity, higher non-motor burden, more depressive symptoms, and worse cognition (in the attention, executive function, memory, and visuospatial domains) than the group where patients and informants agreed. A previous study found patients who underestimated ADL disabilities had shorter disease durations, more preserved cognitive abilities, and were living in a family environment, while those who overestimated their ADL skills had advanced PD, showed cognitive dysfunctions, and lived alone (Shulman et al., 2006). It would be interesting for future studies to determine to what extent difficulties in ADL are results of these factors and the disease progression. Furthermore, studies should explore different factors related to either the patient (such as comorbid diseases, neuropsychiatric disturbances, or other common non-motor symptoms including fatigue/sleep disturbances, or urinary dysfunction) or the informant (personality traits, quality of life, stress, and psychosocial burden) that could influence ADL function, and how these may affect ratings.

There are limitations of this study that need to be addressed. Most importantly, no objective measures of ADL function, such as performance-based tests which were used in Deck et al. (2019), were used to validate the ADL ratings of the FAQ in this study. Therefore, we cannot determine whether patients’ or their informants’ ratings correspond most accurately to the real-life functional performance. While performance-based tests have been used in PD research, these measures are often not feasible in clinical and research settings as they are time-consuming and can be heavily influenced by motor severity (Sulzer et al., 2020). Future studies should incorporate objective measures of ADL function to determine the relation of both patient and informant ratings to objective observed ADL performance and to overcome any methodological biases or inaccuracy errors that may be related to the use of questionnaires (Sadek et al., 2011). Moreover, the current analyses should be repeated using different ADL scales, to determine whether they show better or worse agreement between patients and informants, to aid both clinicians and researchers in their selection of assessment tools. The current results should also be interpreted with caution due to the imbalanced group sizes for each cognitive status (especially for the PDD group). Studies should seek to replicate current findings using not only equal sample sizes, but also using different neuropsychological tests or different cut-offs for diagnosis of cognitive impairment. Furthermore, as briefly mentioned, we did not evaluate more specific caregiver details, as the primary outcome of the study was changes in ADL functioning of patients (with information from informants supporting the evaluation of ADL function). Certain sub-analyses of caregiver variables also could not be performed in this study. Examination of whether relation to and time spent with the patient affected agreement were not possible due to a too-small group sizes for adequately powered analyses, and agreement statistics between different categories of caregiver relationship were unable to be run in certain cases due to variables being constant. More studies are needed to determine the extent that informant factors may have on their perceptions of patient ADL functions.

## Conclusion

This study examined self- and informant-reported ADL using a widely known questionnaire in a cohort of PD patients with varying degrees of cognitive impairment by looking at the agreement between both raters as well as associations with both patient and informant characteristics. Results of the study showed that when using the same ADL questionnaire, there was only a fair to moderate agreement between patients and their informants regarding the patients’ daily functioning, with divergence between ratings increasing as cognitive impairment becomes more severe. Overall, less than one-third (29.3%) of PD patients had perfect agreement with informants on the FAQ total score, highlighting again that this discrepancy is very pronounced. Our results also highlight that motor severity influences ADL ratings and modulates associations to cognition, Lastly, as the diagnosis of PDD necessitates impairments in ADL to be cognitive based, it is important for clinicians to understand that both informants and patients are affected by patient characteristics (most notably motor severity) and must take these into account and rule out their influences when deciding on a dementia diagnosis.

## Data Availability Statement

The datasets presented in this article are not readily available because the corresponding author will consider any requests for access to the data (including de-identified participant data and corresponding data dictionary) reported in this manuscript. Requests to access the datasets should be directed to IL-S, inga.liepelt@uni-tuebingen.de.

## Ethics Statement

The studies involving human participants were reviewed and approved by the Ethics Committee of the Medical Faculty of University of Tübingen. The patients/participants provided their written informed consent to participate in this study.

## Author Contributions

SB, BF, and IL-S: design and conceptualization of study. SB, SS, KM, and KB: data collection. SB and IL-S: statistical analysis. SB: writing of the first draft. SS, KM, BF, KB, and IL-S: review and critique of the manuscript. All authors contributed to the article and approved the submitted version.

## Conflict of Interest

The authors declare that the research was conducted in the absence of any commercial or financial relationships that could be construed as a potential conflict of interest.

## Publisher’s Note

All claims expressed in this article are solely those of the authors and do not necessarily represent those of their affiliated organizations, or those of the publisher, the editors and the reviewers. Any product that may be evaluated in this article, or claim that may be made by its manufacturer, is not guaranteed or endorsed by the publisher.
