# Supplementary material for: Divergence Between Informant and Self-Ratings of Activities of Daily Living Impairments in Parkinson’s Disease
Source: Front Aging Neurosci. 2022 Feb 11;14:838674. doi: 10.3389/fnagi.2022.838674 (PMC8874137; doi:10.3389/fnagi.2022.838674)
Supplement: Supplementary file 1 [file Data_Sheet_1.docx]

Supplementary Table 1*: Agreement between self and informant ratings of the FAQ according to sex of the patient*

|  | Male Patient  *n=94* | | | | | Female Patient  *n=56* | | | | |
| --- | --- | --- | --- | --- | --- | --- | --- | --- | --- | --- |
| FAQ Item | Self | Informant | Weighted *κ* | SE of *κ* | *p*-value | Self | Informant | Weighted *κ* | SE of *κ* | *p*-value |
| 1: Handling finances | 14 (14.9) | 19 (20.2) | 0.62 | 0.10 | <0.001** | 6 (10.7) | 17 (30.4) | 0.55 | 0.11 | <0.001** |
| 2: Assembling tax records | 27 (28.7) | 31 (33) | 0.51 | 0.09 | <0.001** | 20 (35.7) | 25 (44.6) | 0.41 | 0.11 | <0.001** |
| 3: Shopping | 18 (19.1) | 27 (28.7) | 0.52 | 0.10 | <0.001** | 9 (16.1) | 14 (25) | 0.42 | 0.13 | <0.001** |
| 4: Skills and hobbies | 13 (13.8) | 35 (37.2) | 0.30 | 0.08 | <0.001** | 8 (14.3) | 23 (41.1) | 0.23 | 0.11 | 0.008** |
| 5: Using appliances | 9 (9.6) | 14 (14.9) | 0.46 | 0.13 | <0.001** | 3 (5.4) | 10 (17.9) | 0.51 | 0.17 | <0.001** |
| 6: Meal preparation | 28 (29.8) | 38 (40.4) | 0.36 | 0.09 | <0.001** | 3 (5.4) | 14 (25) | 0.34 | 0.15 | <0.001** |
| 7: Current events | 11 (11.7) | 19 (20.2) | 0.47 | 0.12 | <0.001** | 2 (3.6) | 7 (12.5) | 0.10 | 0.11 | 0.30 |
| 8: Paying attention | 15 (16) | 22 (23.4) | 0.40 | 0.09 | <0.001** | 5 (8.9) | 8 (14.3) | 0.39 | 0.15 | <0.001** |
| 9: Remembering appointments | 28 (29.8) | 37 (39.4) | 0.37 | 0.08 | 0.002** | 9 (16.1) | 11 (19.6) | 0.41 | 0.14 | <0.001** |
| 10: Traveling out of house | 15 (16) | 21 (22.3) | 0.68 | 0.09 | <0.001** | 11 (19.6) | 21 (37.5) | 0.51 | 0.11 | <0.001** |

Results are expressed as *Number of patients scoring ≥ 1 (%)*, **p*<0.05, ***p*<0.01

FAQ, Functional Activities Questionnaire; SE, standard error

Supplementary Table 2*: Agreement between self and informant ratings of the FAQ according to disease duration*

|  | Disease Duration ≤7.31 years  *n=75* | | | | | Disease Duration >7.32 years  *n=75* | | | | |
| --- | --- | --- | --- | --- | --- | --- | --- | --- | --- | --- |
| FAQ Item | Self | Informant | Weighted *κ* | Standard Error of *κ* | *p*-value | Self | Informant | Weighted *κ* | Standard Error of *κ* | *p*-value |
| 1: Handling finances | 5 (6.7) | 17 (22.7) | 0.53 | 0.13 | <0.001** | 15 (20) | 19 (25.3) | 0.62 | 0.09 | <0.001** |
| 2: Assembling tax records | 20 (26.7) | 28 (37.3) | 0.48 | 0.10 | <0.001** | 27 (36) | 28 (37.3) | 0.46 | 0.09 | <0.001** |
| 3: Shopping | 12 (16) | 17 (22.7) | 0.51 | 0.13 | <0.001** | 15 (20) | 24 (32) | 0.46 | 0.10 | <0.001** |
| 4: Skills and hobbies | 8 (10.7) | 30 (40) | 0.22 | 0.08 | 0.01* | 13 (17.3) | 28 (37.3) | 0.33 | 0.10 | <0.001** |
| 5: Using appliances | 4 (5.3) | 8 (10.7) | 0.62 | 0.15 | <0.001** | 8 (10.7) | 16 (21.3) | 0.39 | 0.15 | <0.001** |
| 6: Meal preparation | 14 (18.7) | 24 (32) | 0.53 | 0.10 | <0.001** | 17 (22.7) | 28 (37.3) | 0.24 | 0.11 | 0.007** |
| 7: Current events | 6 (8) | 9 (12) | 0.39 | 0.17 | <0.001** | 7 (9.3) | 17 (22.7) | 0.35 | 0.13 | <0.001** |
| 8: Paying attention | 10 (13.3) | 11 (14.7) | 0.35 | 0.13 | <0.001** | 10 (13.3) | 19 (25.3) | 0.44 | 0.09 | <0.001** |
| 9: Remembering appointments | 18 (24) | 20 (26.7) | 0.31 | 0.10 | <0.001** | 19 (25.3) | 28 (37.3) | 0.46 | 0.09 | <0.001** |
| 10: Traveling out of house | 10 (13.3) | 23 (30.7) | 0.50 | 0.11 | <0.001** | 16 (21.3) | 19 (25.3) | 0.69 | 0.09 | <0.001** |

Results are expressed as *Number of patients scoring ≥ 1 (%)*, **p*<0.05, ***p*<0.01

FAQ, Functional Activities Questionnaire

Supplementary Table 3*: Agreement between self and informant ratings of the FAQ according to depressive symptomatology*

|  | BDI-II score ≤9  *n=79* | | | | | BDI-II score >10  *n=71* | | | | |
| --- | --- | --- | --- | --- | --- | --- | --- | --- | --- | --- |
| FAQ Item | Self | Informant | Weighted *κ* | Standard Error of *κ* | *p*-value | Self | Informant | Weighted *κ* | Standard Error of *κ* | *p*-value |
| 1: Handling finances | 4 (5.1) | 11 (13.9) | 0.52 | 0.16 | <0.001** | 16 (22.5) | 25 (35.2) | 0.59 | 0.09 | <0.001** |
| 2: Assembling tax records | 21 (26.6) | 22 (27.8) | 0.62 | 0.10 | <0.001** | 26 (36.6) | 34 (47.9) | 0.35 | 0.09 | <0.001** |
| 3: Shopping | 7 (8.9) | 14 (17.7) | 0.52 | 0.13 | <0.001** | 20 (28.2) | 27 (38.0) | 0.43 | 0.10 | <0.001** |
| 4: Skills and hobbies | 7 (8.9) | 20 (25.3) | 0.41 | 0.12 | <0.001** | 14 (19.7) | 28 (53.5) | 0.17 | 0.08 | 0.02* |
| 5: Using appliances | 2 (2.5) | 5 (6.3) | 0.66 | 0.18 | <0.001** | 10 (14.1) | 19 (26.8) | 0.40 | 0.13 | <0.001** |
| 6: Meal preparation | 11 (13.9) | 27 (34.2) | 0.44 | 0.10 | <0.001** | 20 (28.2) | 25 (35.2) | 0.32 | 0.11 | <0.001** |
| 7: Current events | 4 (5.1) | 7 (8.9) | 0.40 | 0.21 | <0.001** | 9 (12.7) | 19 (26.8) | 0.34 | 0.12 | <0.001** |
| 8: Paying attention | 6 (7.6) | 10 (12.7) | 0.39 | 0.15 | <0.001** | 14 (19.7) | 20 (28.2) | 0.37 | 0.10 | <0.001** |
| 9: Remembering appointments | 15 (19) | 18 (22.8) | 0.31 | 0.12 | <0.001** | 22 (31) | 30 (42.3) | 0.42 | 0.09 | <0.001** |
| 10: Traveling out of house | 7 (8.9) | 12 (15.2) | 0.72 | 0.11 | <0.001** | 19 (26.8) | 20 (42.3) | 0.52 | 0.10 | <0.001** |

Results are expressed as *Number of patients scoring ≥ 1 (%)*, **p*<0.05, ***p*<0.01

BDI, Beck Depression Inventory-II; FAQ, Functional Activities Questionnaire; SE, standard error
